# Gut microbiota modulate immune responses to orally and parenterally administered rotavirus in mice

**DOI:** 10.1038/s41541-025-01126-9

**Published:** 2025-04-20

**Authors:** Zhenda Shi, Sung-Sil Moon, Jun Zou, Yanling Wang, Noopur Bhatnagar, Vu L. Ngo, Xiaoqian Wang, Houping Wang, Theresa K. Bessey, Jennifer J. Hull, Yuhuan Wang, Sang-Moo Kang, Andrew T. Gewirtz, Baoming Jiang

**Affiliations:** 1 Division of Viral Diseases, National Center for Immunization and Respiratory Diseases, Centers for Disease Control and Prevention, Atlanta, GA USA; 2 Cherokee Nation Operational Solutions, Cherokee Federal, Atlanta, GA and, Tulsa, OK USA; 3 Institute for Biomedical Sciences, Georgia State University, Atlanta, GA USA

**Keywords:** Adjuvants, Translational research

## Abstract

Rotavirus (RV) remains a significant cause of infantile morbidity and mortality, while oral RV vaccines offer inconsistent protection. This study investigates whether gut microbiota influence immune responses to orally and intramuscularly (IM) administered RV strains. Using murine models, we identified microbiota constituents, including segmented filamentous bacteria, reducing oral RV infection and RV antibody generation. Such blockade of RV-induced responses was associated with elevated expression of intestinal Reg3β and Reg3γ and was recapitulated by intraperitoneal administration of cognate recombinant proteins. IM administration following oral RV inoculations enhanced antibody production and defense against RV challenge. We further showed microbiota composition also influenced the efficacy of a single IM RV inoculation. Antibiotic-induced microbiota depletion boosted IM RV efficacy in poorly responding animals. Such enhancement of IM RV-induced immunity appeared to be associated with increased expression of serum RANTES and Eotaxin. The phenotype was recapitulated by directly adjuvating these chemokines to the IM inoculum.

## Introduction

Rotavirus (RV) is a highly contagious, non-enveloped double-stranded RNA virus responsible for severe diarrhea in infants and young children worldwide^1^. Despite extensive efforts, no specific anti-RV drugs exist. Vaccines play a crucial role in preventing RV infections and reducing disease severity^2,3^. While oral vaccines like Rotarix^®^ and RotaTeq^®^ have been effective in high-income regions, suboptimal coverage persists. Approximately 17% of vaccinated individuals in these regions remain unprotected. Additional oral vaccines, such as Rotavac^®^ and Rotasiil^®^, have been introduced in low- and middle-income regions; however, their efficacies remain significantly lower, with failure rates reaching 46%^2,4,5^. The mechanisms underlying this variability warrant further investigation and understanding the factors influencing vaccine efficacy is critical to improve public health outcomes and guide future vaccine development efforts.

The current understanding of the anti-viral effects of bacteria against eukaryotic viruses (non-phage) remains limited^6^. Recent studies suggest that bacteria can either facilitate or inhibit viral infections through various mechanisms, highlighting the critical role of gut microbiota in determining host susceptibility to viral infections^7–10^. Segmented filamentous bacteria (SFB), known for their ability to mediate mucosal immunity and induce Th17 responses^11^, have been shown to suppress RV infection in mice, independent of the acquired immune system^12^. This leads us to hypothesize that SFB-containing microbiota might interfere with the effectiveness of live attenuated RV vaccines. However, the composition of mouse microbiota can significantly vary across different animal facilities, even among mice of the same genotype but housed under varied conditions^3^. Such variability complicates the interpretation and replication of research findings. To address this challenge, we employed two distinct mouse models: excluded flora (EF) and murine pathogen-free flora (MPF) C57BL/6 mice from Taconic Biosciences. These models provide genetically identical mice with differing microbiota compositions—MPF mice are colonized with SFB, while EF mice lack SFB colonization^13^. We analyzed EF and MPF WT mice responses to model RV inoculations, investigating the impact of modulating microbiota and administering the inoculum via the intramuscular (IM) route. Select microbiota, influenced by antibiotic treatment, significantly affected the efficacy of orally and intramuscularly administered RV vaccines. The dampened oral RV inoculation efficacy correlated with increased expression of intestinal Reg3β and Reg3γ and the phenotype was replicated through the direct administration of corresponding recombinant proteins. Furthermore, our results suggested approaches to recapitulate microbiota-induced immune signaling, particularly via chemokines RANTES and Eotaxin, as potential adjuvants to increase IM RV vaccine efficacy without directly manipulating microbiota.

## Results

### Microbiota composition influences infection and antibody response to oral RV inoculation

**Fig. 1:**
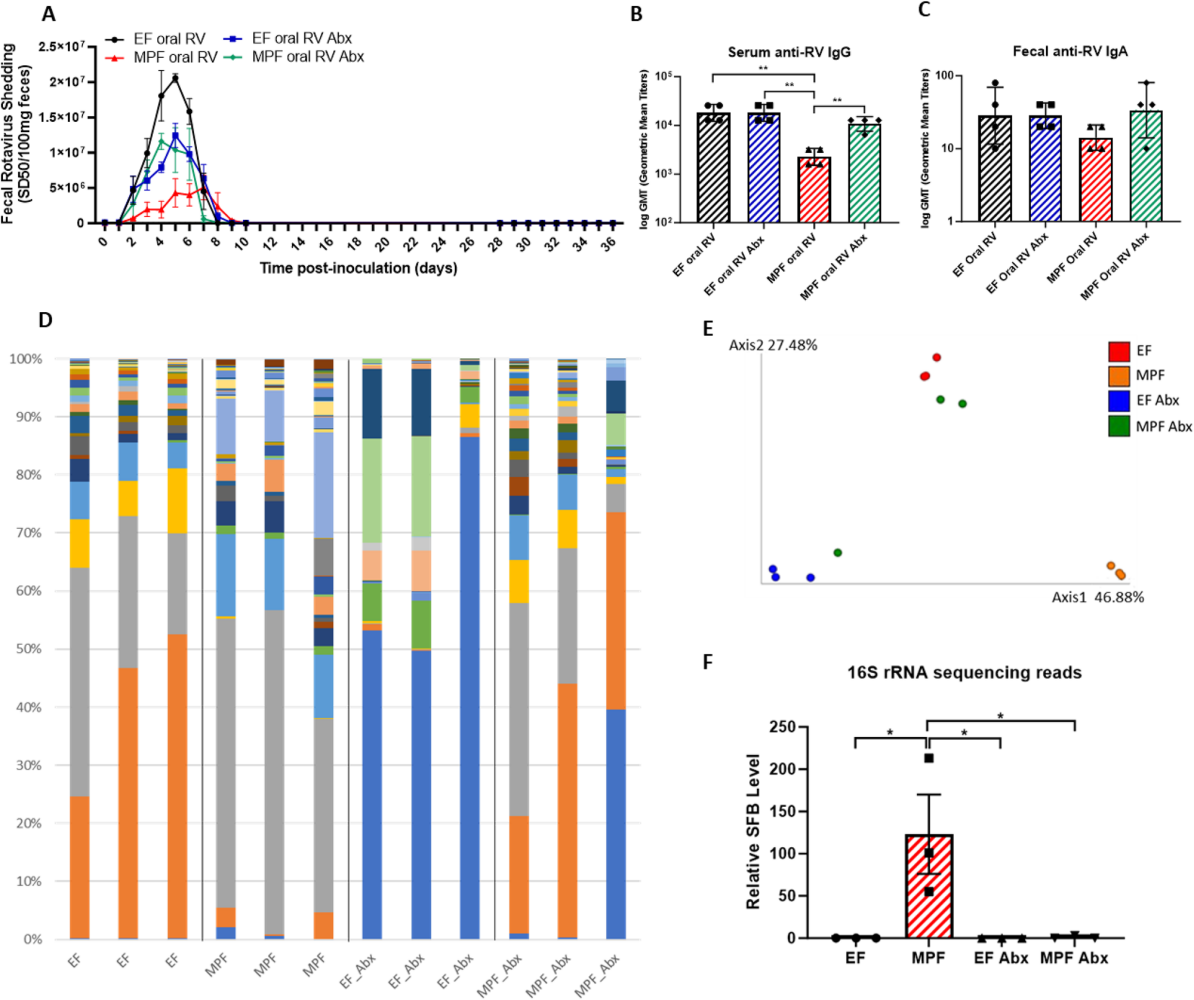
Microbiota composition influences infection and antibody response to oral RV inoculation. EF or MPF mice were treated with or without antibiotics (Abx) for one week before oral RV inoculation (red arrow) and throughout the experiment. Microbiota compositions were analyzed on 0 dpi using 16S-rRNA sequencing. Serum and fecal samples were collected on 21 dpi; serum RV-specific IgG and fecal RV-specific IgA levels were assayed using ELISA. The mice were then challenged orally with RV (red lightning bolt) on 28 dpi and fecal RV shedding was assayed by ELISA. **A** Fecal RV antigen shedding. Data are shown as means ± SEM. RV shedding differed significantly among the 4 groups, except the EF oral RV Abx group and the MPF oral RV Abx group (*n* = 4, *p* < 0.0001, two-way ANOVA). **B** Serum RV-specific IgG levels. Data are shown as geometric mean titer (GMT). The MPF oral RV group differed significantly from the other 3 groups (*n* = 4, ** *p* < 0.01, T-test). The other 3 groups showed no statistical significance. **C** Fecal RV-specific IgA levels. Data are shown as GMT. No statistical significance was found among the 4 groups (*n* = 4). **D** Microbiota compositions: Analyzed for EF and MPF mice with/without antibiotics (*n* = 3) . **E** Principal component analysis (PCA) plot of microbiota. Axis1 represents a 46.88% difference and Axis2 represents a 27.48% difference among the 4 groups (*n* = 3). **F** SFB 16S-rRNA sequence reads. Data are shown as means ± SEM (*n* = 3, *p* < 0.05, T-test). Each experiment was performed two times and yielded an identical pattern of results.

Administering RV to adult mice results in asymptomatic infection, generating anti-RV antibodies preventing subsequent RV infection, serving as a model for understanding RV vaccination determinants^14^. WT C57BL/6 mice, EF (SFB free) or MPF vivarium (SFB-positive)^13^ were orally administered murine RV (EC strain, same amount of 10^4^–10^5^ Shedding dose 50% (SD_50_)). Fecal samples were collected daily for 10 days post-inoculation (dpi) and showed significantly lower RV antigen shedding in MPF mice compared to EF mice (Fig. 1A), assayed using enzyme-linked immunoassay (ELISA). Analysis of blood and fecal samples on day 21 showed lower serum anti-RV IgG and fecal anti-RV IgA levels in the MPF mice, with a statistically significant difference in serum but not in feces. (Fig. 1B, C). After four-weeks, a homologous oral RV challenge was performed. Despite showing varying levels of RV antigen shedding and having lower RV-specific IgG levels after the initial oral RV inoculation, both EF and MPF mice were fully protected, as evidenced by the absence of detectable fecal RV antigens (Fig. 1A). In parallel, we investigated the role of gut microbiota modulated immune responses to RV inoculation in genetically identical hosts. We administered antibiotics (ampicillin, neomycin, streptomycin, and metronidazole) to both EF and MPF mice, starting one week before RV inoculation and maintained throughout the experiment. Notably, the antibiotic treatment led to reduced RV antigen shedding in EF mice, aligning with our previous research findings^10^. In contrast, antibiotic treatment increased RV antigen shedding in the MPF mice, suggesting that the MPF mice microbiota had suppressed RV infection. Four-weeks post RV inoculation, these mice received a homologous oral RV challenge. Regardless of their microbiota compositions or whether they received antibiotic treatment, the mice were all fully protected (Fig. 1A). Moreover, antibiotic treatment did not alter RV-specific IgG levels in EF mice, whereas it significantly increased the IgG levels in MPF mice to a level similar to that of EF mice (Fig. 1B). We next defined the microbiota composition of EF and MPF mice with and without the antibiotic treatment by 16S rRNA sequencing. Microbiomes of EF and MPF mice differed significantly, and antibiotic treatment shifted microbiota composition of both EF and MPF mice, causing the microbiomes of the treated MPF mice to resemble those of the treated and untreated EF mice. (Fig. 1C, D). The level of SFB, a mediator of the host immune response present in MPF but not EF mice, was markedly reduced following the antibiotic treatment (Fig. 1E). Additionally, the relative abundance of other microbiota taxa in MPF mice was significantly altered by antibiotics treatment compared to the other three groups. These taxa include *Oscillospiraceae, Butyricimonas, Desulfovibrionaceae, Rikenellaceae*, and P*eptococcaceae*. Detailed information on microbiota composition is provided in Supplementary Fig. 1.

### Combination treatment of Reg3β and Reg3γ inhibits RV infection in WT and Rag1-KO mice

**Fig. 2:**
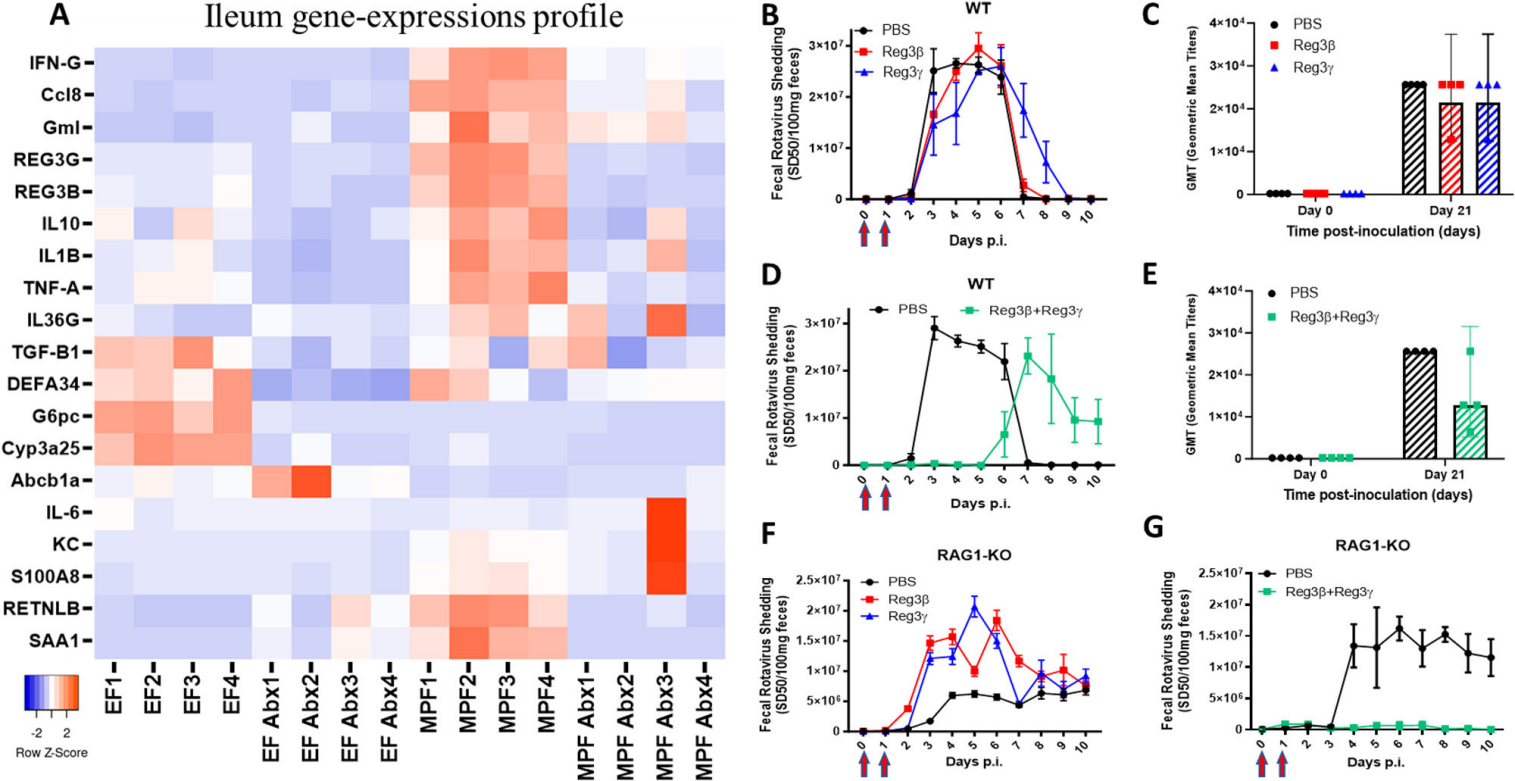
Combination treatment of Reg3β and Reg3γ inhibits RV infection in WT and Rag1-KO mice. **A** Heatmap of ileum cytokine expression levels. EF and MPF mice were treated with or without antibiotics for one week. Ileum mRNA was then extracted, and representative pro-inflammatory and anti-inflammatory cytokine expression levels were analyzed using qPCR (*n* = 4). **B–G** WT or Rag1-KO mice were intraperitoneally (IP) injected with or without Reg3β (10 µg), Reg3γ (10 µg), or both on 0 dpi and 1 dpi (red arrows). The treated mice were challenged with RV orally on 0 dpi. Feces were collected daily for 10 days, and RV antigen shedding was quantified by ELISA. Serum was collected on 0 dpi and 21 dpi, and RV-specific IgG levels were assayed by ELISA. **B**, **C** WT mice received IP treatment of PBS, Reg3β, or Reg3γ. **B** Fecal RV antigen shedding. **C** Serum RV-specific IgG on 0 dpi and 21 dpi. **D**, **E** WT mice treated with PBS or combined Reg3β and Reg3γ. **D** Fecal RV antigen shedding. **E** Serum RV-specific IgG level on 0-dpi and 21-dpi. **F**, **G** Rag1-KO mice treated with PBS, Reg3β, or Reg3 γ independently (**F**) or both (**G**). Results shown in Fig. (**B**, **D**, **F**, **G**) are mean ± SEM (*n* = 4). (**D**, **G**), combined treatment of Reg3β and Reg3γ group differed from the PBS group significantly (*n* = 4, *p* < 0.0001, two-way ANOVA). Each experiment was performed 2 times and yielded an identical pattern of results.

We next investigated potential mechanisms by which microbiota might impact shedding levels of RV and, subsequently, host’s immune responses following oral RV inoculation. We first investigated host correlates of the response by probing expression levels of an array of pro-inflammatory and anti-inflammatory genes^15^, whose expression differed in the intestines of the EF and MPF mice with or without antibiotics treatment by real-time PCR. The results showed that certain genes, including Reg3β and Reg3γ, were significantly up regulated in the ileum of the MPF mice in comparison with the EF mice, and EF and MPF mice with antibiotics treatment (Fig. 2A and Supplementary Fig. 2A). To probe the potential involvement of these genes in mediating the anti-RV effect, we intraperitoneally (IP) injected WT mice with Reg3β, Reg3γ, or combined, on 0 dpi and 1 dpi of oral RV inoculation. Results showed that independent treatment with either recombinant protein did not inhibit the mice from RV infection (Fig. 2B) nor were 21 dpi serum RV-specific IgG levels were significantly affected (Fig. 2C). In contrast, the combined treatment of Reg3β and Reg3γ significantly delayed RV infection in WT mice (Fig. 2D). Serum RV-specific IgG level trended lower in mice receiving the combination treatment (Fig. 2E). Other genes, including Retnlb and SAA1 were also tested in the same manner, but did not show any significant effect (Supplementary Fig. 2B). IFN-γ was not tested due to we previously ruled out the involvement of IFN-γ in SFB mediated anti-RV effect using neutralizing antibody against IFN-γ^12^. We further investigated whether this protective effect against RV challenge was dependent or independent of the host acquired immune system using Rag1-KO mice, which lack acquired immune systems^16^. Results showed that independent treatment with Reg3β or Reg3γ did not significantly protect against RV infection in Rag1-KO mice (Fig. 2F). However. the combined treatment of Reg3β and Reg3γ fully suppressed RV infection in the immunodeficient mice (Fig. 2G).

### The addition of IM RV administration enhances the defense of oral RV vaccine non-responders against oral RV challenge

**Fig. 3:**
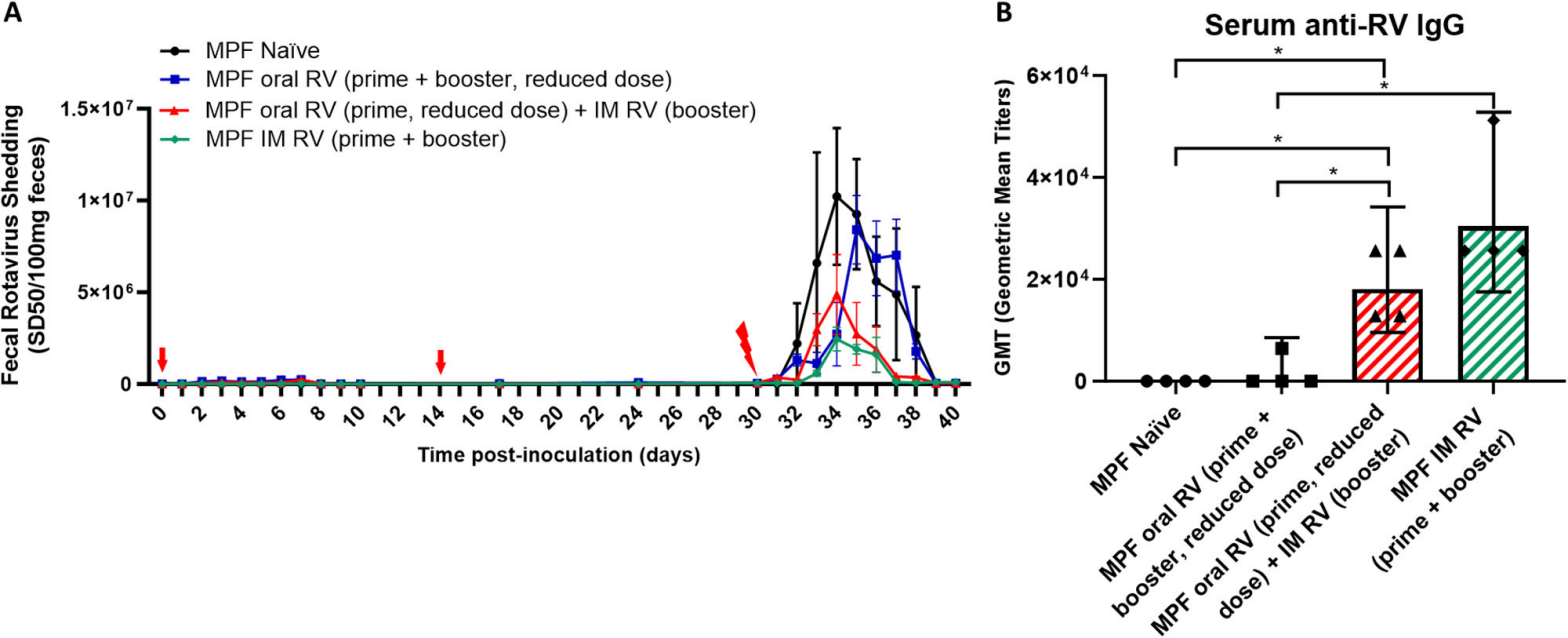
The addition of IM RV administration enhances the defense of oral RV vaccine non-responders against oral RV challenge. MPF mice were orally inoculated with reduced dose of RV or IM inoculated with RV twice on 0 dpi and 14 dpi (prime + booster, red arrows) or given an oral RV inoculation (prime) on 0 dpi and an IM RV inoculation (booster) on 14 dpi. Oral inoculation used 10 SD_50_ RV EC strain, and IM inoculation used 5 μg live RV Wa strain. Naïve MPF mice did not receive any inoculation. Serum was collected on 28 dpi, and RV-specific IgG levels were assayed by ELISA. The mice were challenged orally with RV on 30 dpi. Fecal samples were collected on indicated days, and RV antigen shedding was assayed using ELISA. **A** Fecal RV antigen shedding. **B** Serum RV-specific IgG levels. Data shown in (**A**) are means ± SEM, oral RV (prime) + IM RV (booster) group and IM RV (prime + booster) group differed naïve and oral RV (prime + booster) groups significantly (*n* = 4, *p* < 0.0001, two-way ANOVA). Data shown in (**B**) are GMT (*n* = 4, *P < 0.05, T-test). Each experiment was performed 2 times and yielded an identical pattern of results.

The interference of some microbiota on generation of RV antigens and antibodies in response to oral RV inoculation prompted us to use MPF mice as a model to examine IM RV (Wa strain) administration as primary immunization series or as a booster dose to oral immunization to improve host immune defense against RV challenge. For this purpose, we used a reduced oral inoculation dose (10 SD_50_, EC strain) to mimic the scenario of oral RV vaccine non-responders. MPF mice were administered with two oral RV doses, one oral RV dose and one IM dose of RV inoculations, or two IM RV doses followed by homologous oral challenge with RV. Mice that were administered two oral doses, one oral plus one IM dose, or two IM doses did not exhibit RV antigen shedding (Fig. 3A). When orally challenged, the group that received oral inoculation alone failed to suppress RV antigen shedding, while mice that received one oral plus one IM RV inoculation or two IM doses showed significant reductions in RV-specific antigen shedding compared to the naïve and the orally inoculated groups (Fig. 3A). Additionally, oral inoculation with reduced RV doses did not lead to the induction of RV-specific IgG in the serum. In contrast, a combination of oral and IM RV inoculations, as well as two doses of IM RV inoculations, significantly elevated serum RV-specific IgG levels in the MPF WT mice (Fig. 3B).

### Host microbiota affect parenteral RV vaccination

**Fig. 4:**
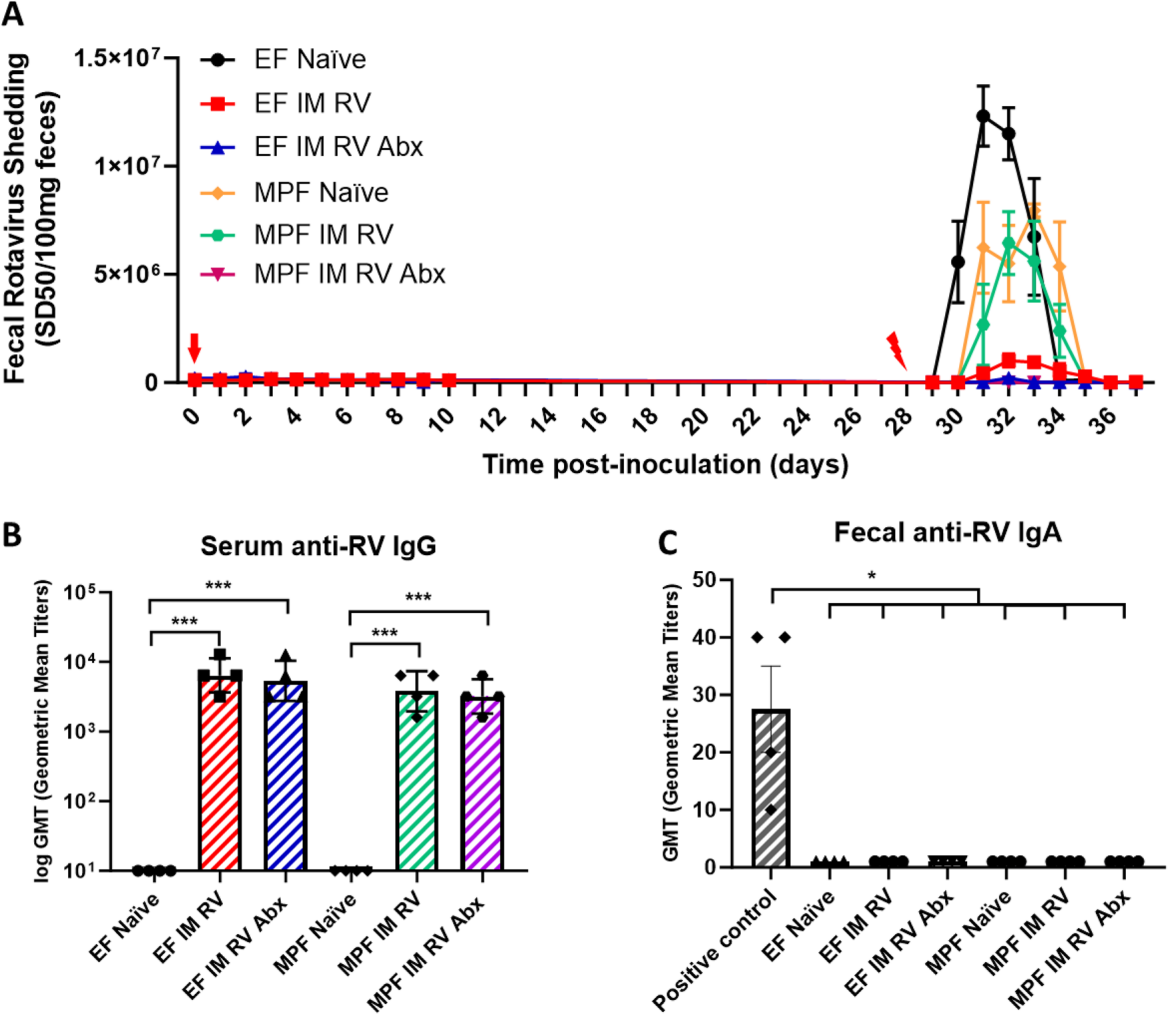
Host microbiota affect parenteral RV vaccination. EF and MPF mice were treated with or without antibiotic cocktail in drinking water for one week prior to IM RV inoculation (Wa strain 5 μg live, red arrow) and maintained throughout the experiment. Serum and fecal samples were collected on 21 dpi and serum RV-specific IgG and fecal RV-specific IgA levels were assayed respectively using ELISA. Positive fecal IgA control used previously oral RV (EC strain) inoculated mice fecal samples. Naïve mice were not inoculated or treated with antibiotics. The mice were challenged orally (red lightning bolt mark) with RV on 28 dpi. Fecal samples were collected on the indicated days, and RV antigen shedding was assayed using ELISA. **A** Fecal RV antigen shedding. **B** Serum RV specific IgG levels. **C** Fecal RV specific IgA levels. Data shown in (**A**) are means ± SEM, EF IM RV differed from all other groups significantly, EF IM RV Abx and MPF IM RV Abx groups differed from all the other groups significantly (*n* = 4. *p* < 0.0001, two-way ANOVA). MPF naïve group differed from EF naïve group significantly. Data shown in (**B**, **C**) are GMT (*n* = 4, *p < 0.05; ***p < 0.001. T-test). No significance was found in (**B**) among the four IM RV inoculated groups, or in (**C**) among the groups excluding the positive control. Each experiment was conducted twice and yielded consistent results.

We next examined impacts of microbiota on parenteral RV administration. Results showed that either EF or MPF mice that were IM injected with purified RV (Wa strain live, 5 μg) did not shed RV-antigen (Fig. 4A). Both EF and MPF mice generated similar levels of serum RV-specific IgG (Fig. 4B), and the one-time IM RV inoculation induced very limited fecal RV-specific IgA in both strains of mice (Fig. 4C). Nonetheless, despite showing similar levels of serum RV-specific IgG and little fecal RV specific IgA, the MPF mice that received IM RV immunization were significantly less protected from the challenge compared to the EF mice (Fig. 4A). To understand whether this phenotype was RV strain-specific, we repeated the IM RV immunization experiment using a simian RV (RRV) as the injection antigen. The results showed similar outcomes that EF mice were fully protected with no detectable fecal RV antigen shedding from RV challenge, while MPF mice were significantly less protected. This suggests that the reduced protection from RV challenge following IM RV immunization in MPF mice is not RV strain-specific (Supplementary Fig. 3). In addition, to understand whether this phenotype is microbiota dependent, we treated EF and MPF mice with antibiotics in drinking water starting one-week prior to IM RV inoculation, and performed RV oral challenges, measured fecal RV shedding and serum RV-specific IgG levels. The results showed that the antibiotic treatment modestly reduced RV shedding in EF mice from RV challenge but significantly enhanced the protection against oral RV challenge in the MPF mice, as exhibited with little RV antigen shedding detected (Fig. 4A). Despite significantly improved protection, the administration of antibiotics did not alter serum RV-specific IgG and fecal RV-specific IgA levels in the IM-inoculated EF and MPF mice; positive fecal IgA control used previously oral inoculated mice fecal samples. (Fig. 4B, C). Subsequently, we examined the functional capacity of antibodies elicited by these IM inoculations through RV neutralization assays. RV-specific antibody levels did not differ significantly in neutralizing capabilities among EF and MPF mice that treated with or without antibiotics. This argues against the hypothesis that the dampened protection against RV challenge in MPF mice without antibiotic treatment was dependent on neutralizing activity (Supplementary Fig. 4).

We next investigated the potential of microbiota to modulate another injectable vaccine, the influenza vaccine. EF and MPF mice received drinking water with or without antibiotics, starting 1-week before vaccination. Then, the mice were IM immunized with inactivated split A/California/04/2009 (A/Cal) H1N1 Influenza Vaccine (sCal) on 0 dpi, and intranasally challenged with 3X LD50 A/Cal on 21 dpi^17^. Body weight changes were monitored, and mice that experienced a loss of ≥ 20% of their initial body weight were sacrificed and removed from experiment. The naïve EF, naïve MPF, and MPF mice that received only the sCal vaccine, showed severe weight loss and were removed from the experiment between 9-dpi and 12-dpi after challenge. The sCal vaccine significantly protected the vaccinated EF mice, irrespective of antibiotics treatment, compared to the naïve mice. In contrast, antibiotics induced significantly better protection against A/Cal virus challenge in sCal vaccinated MPF mice, as indicated by delayed and attenuated reduction in body weight losses (Supplementary Fig. 5A). Serum A/Cal specific IgG level assay revealed that, despite difference in microbiota composition and antibiotic treatment, there was no statistical significance among the vaccinated groups (Supplementary Fig. 5B), thus indicating that our observations re RV may be applicable to other vaccines.

### Serum cytokine profiles of EF and MPF mice with or without antibiotics treatment

**Fig. 5:**
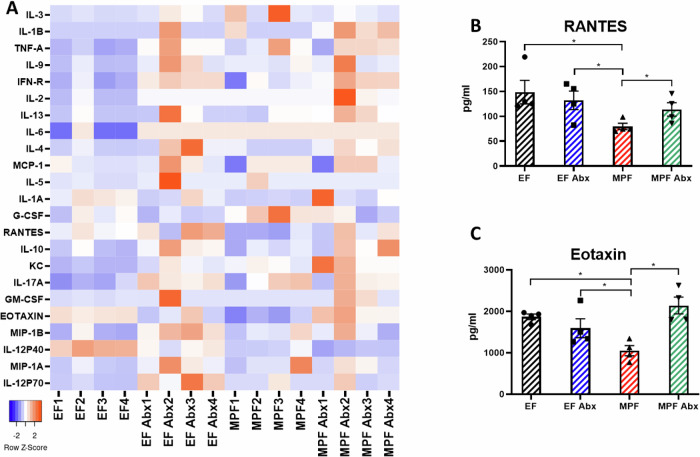
Serum cytokine profiles of EF and MPF mice with or without antibiotics treatment. EF and MPF mice were treated with or without antibiotics in drinking water for one week, followed by analysis of serum cytokine profiles. **A** Heatmap of serum cytokine levels (*n* = 4). **B**, **C** Specific alterations in serum of MPF mice that treated with antibiotics compared to other groups. (**B**) RANTES; (**C**) Eotaxin. (*n* = 4, *, *p* < 0.05, determined by T-test). Each experiment was conducted twice and yielded identical pattern of results.

Among the EF and MPF mice that were treated with or without antibiotics, the MPF group without antibiotic treatment exhibited a dampened efficacy of IM RV inoculation. To understand whether specific cytokines are associated with this phenotype, we interrogated the serum cytokine profiles of naïve EF and MPF mice that treated with or without antibiotics (Fig. 5A). The antibiotic treatment significantly altered serum RANTES, Eotaxin, and IL-12p40 levels in the MPF mice, compared to the other three groups (Supplementary Fig. 6). The alteration of IL-12p40 levels in the MPF mice showed opposite direction of the EF mice treated with or without antibiotics, suggests that this cytokine might not play a role in mediating the IM inoculation efficacy. Conversely, antibiotic treatment significantly elevated serum RANTES and Eotaxin levels in MPF mice, resembling levels observed in both EF mice treated with and without antibiotics (Fig. 5B, C).

### The addition of RANTES and Eotaxin boosts the efficacy of IM RV inoculation against oral RV challenge

**Fig. 6:**
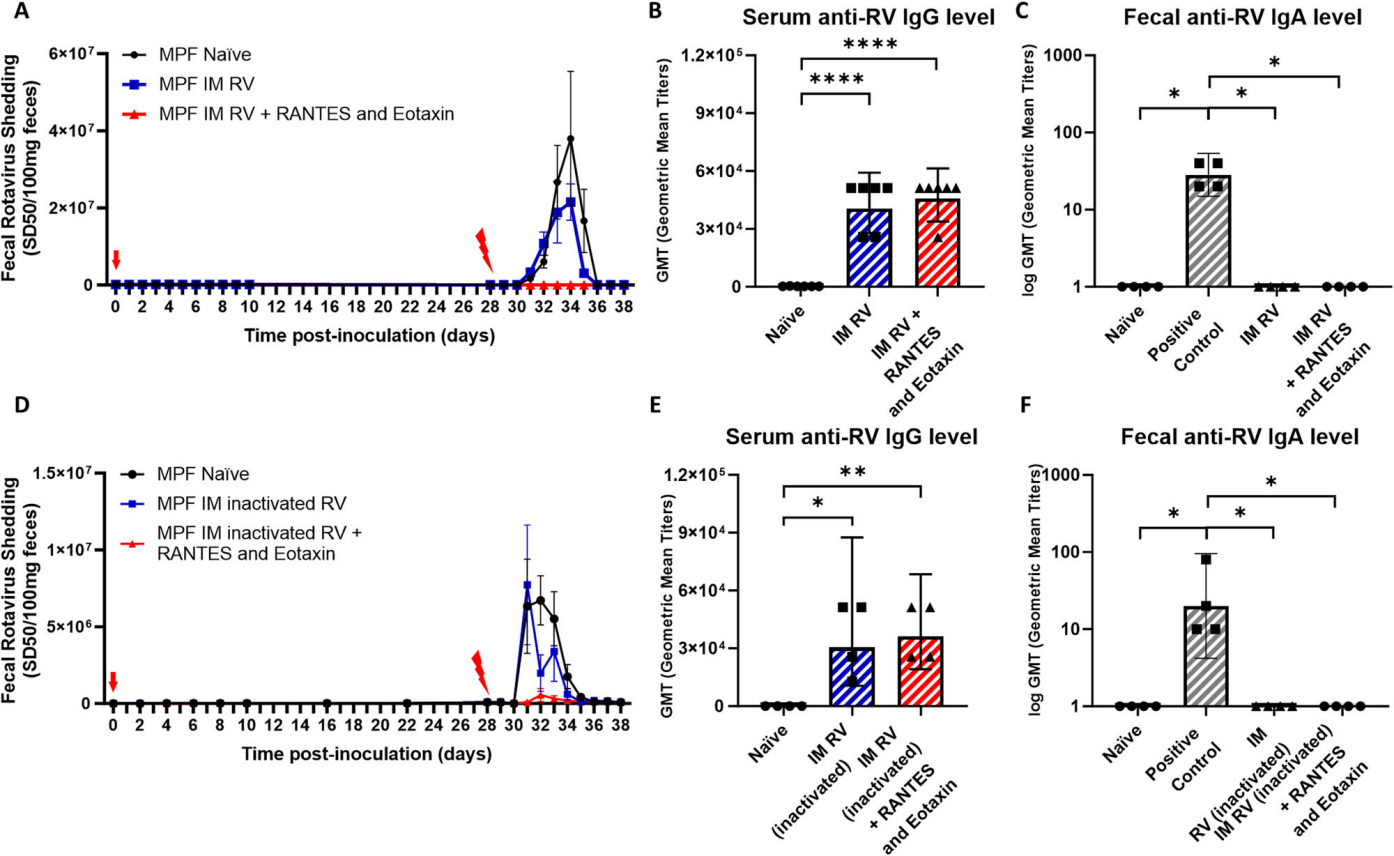
The addition of RANTES and Eotaxin boosts the efficacy of IM RV inoculation against oral RV challenge. MPF mice were IM inoculated with live RV (**A**–**C**) or inactivated RV (**D**–**F**) (Wa strain 5 μg, red arrow), either adjuvanted with or without recombinant RANTES (5 μg) and Eotaxin (2 μg) on 0 dpi and challenged orally with RV (EC strain, red lightning bolt) on 28 dpi. Serum and fecal samples were collected on 21 dpi and serum RV-specific IgG and fecal RV-specific IgA levels were assayed respectively using ELISA. Positive fecal IgA control used previously oral RV (EC strain) inoculated mice fecal samples. Naïve MPF mice did not receive IM inoculation. Fecal samples were collected on the indicated days, and RV antigens shedding was measured using ELISA. **A**, **D** Fecal RV antigen shedding. **B**, **E** Serum RV-specific IgG levels. **C**, **F** Fecal RV-specific IgA levels. Data shown in (**A**) are means ± SEM, all 3 groups differed each other significantly (*n* = 6, *p* < 0.01, two-way ANOVA). Data shown in (**B**–**E**) are GMT (**B**, *n* = 6, ****p < 0.0001; **C**, **D**, **E**, *n* = 4, *, *p* < 0.05, T-test). IM experiments using live RV were performed 2 times and yielded an identical pattern of results; IM experiments using inactivated RV were performed 1 time.

The cytokine results led us to hypothesize that administration of exogenous RANTES and Eotaxin might enhance the efficacy of IM RV inoculation. To test this notion, MPF mice were IM inoculated with either live or inactivated RV (Wa strain, 5 μg) on 0-dpi with or without RANTES and Eotaxin and challenged orally with RV (EC strain, same amount of 10^4^–10^5^ SD_50_) on 28-dpi. Fecal RV antigens shedding, serum RV-specific IgG, and fecal RV-specific IgA levels were assayed. Such direct administration of RANTES and Eotaxin as adjuvants to IM RV immunization did not alter serum RV-specific IgG or fecal RV-specific IgA levels, regardless of the use of live or inactivated RV inoculum, positive fecal IgA control used previously oral RV (EC strain) inoculated mice fecal samples (Fig. 6B, C and Fig. 6E, F). Nonetheless, adjuvating IM RV inoculum, both live and inactivated, with RANTES and Eotaxin significantly improved the efficacy of such inoculation and fully suppressed RV antigen shedding from RV challenge (Fig. 6A, D).

## Discussion

The diminished efficacy of RV vaccines in certain populations has been attributed to factors like poor sanitation, inadequate nutrition, and microbiota variation^18^. Focusing on the latter, our results show an impact of host microbiota on RV inoculation efficacies. Specifically, modulating the microbiota of genetically identical EF and MPF WT mice through antibiotic treatment significantly affected RV-specific IgG levels in serum and RV shedding in the gut. Notably, specific microbiota components, including SFB, played a role in modulating RV resistance. Gene expression profiling revealed up-regulated genes, particularly Reg3β and Reg3γ. Combining Reg3β and Reg3γ treatment significantly delayed and inhibited RV infection, independent of acquired immunity. These findings suggest that manipulating Reg3β and Reg3γ could potentially treat or prevent RV infection, especially in immune-deficient patients. We hypothesized that IM injection of RV could enhance host immune responses to oral RV inoculation in resistant MPF mice, compensating for the dampened antibody response caused by gut microbiota. Our results revealed that adding IM RV injection to oral RV inoculation significantly increased serum RV-specific IgG levels in the MPF mice, thereby protecting them from RV challenge. This combined approach—using both oral and injectable RV vaccines—holds promise for improving RV vaccine effectiveness in children, especially in low-income regions where RV-resistant microbiota prevail.

The investigation of whether host microbiota could modulate IM inoculations revealed that despite inducing similar RV-specific IgG levels in the serum of the host with different microbiota, variations in protection rates were observed. Specifically, we found that MPF mice colonized with the immuno-modulator SFB showed reduced protection against RV challenge following IM RV inoculation compared to EF mice. The antibiotic treatment upon IM RV inoculation gained the MPF mice full capability of defending against RV challenge, indicating the phenotype is microbiota dependent. These findings suggest that microbiota impact the host immune response to RV inoculation beyond the gastrointestinal tract, extending to peripheral systems. Despite bypassing the local gastrointestinal immune system, IM RV administration still exhibits some reduced efficacy in MPF mice, highlighting the broad influence of microbiota. Hence, we propose that microbiota modulation extends to other injectable vaccines like influenza. Prior research shows compromised influenza vaccine efficacy in low- and middle-income countries due to prevalent microbiota^19–21^. Our observation of antibiotics improving influenza vaccine effectiveness in MPF mice aligns with enhanced efficacy seen in injectable RV inoculations, suggesting microbiota could modulate IM vaccines, including influenza.

The mechanism(s) by which microbiota influence immune activity in the peripheral system are not known and may involve serum levels of RANTES and Eotaxin that reduced levels of these chemokines in the host and potentially led to diminished protection against RV challenge. Compensating RANTES and Eotaxin to the IM RV inoculum did not alter the serum RV-specific IgG levels, but fully protected the recipients from the heterologous RV challenge. While both of the chemokines could attract immune cells into sites, RANTES was given a higher dose due to its broader immune-modulating role compared to Eotaxin, which is more eosinophil-specific^22–24^. Future studies will focus on titrating the optimal doses of both cytokines to assess their individual and combined effects on the observed phenotype. Current injectable vaccines have limitations in inducing secretory IgA production and effectively preventing heterologous challenges^25,26^. Adjuvating vaccines with RANTES and Eotaxin might be a potentially novel means for enhancing efficacies of injectable RV vaccines.

While previous studies explored potential correlations between specific microbiota and RV vaccine efficacy^27^, a few have conclusively identified microbiota composition as a determinant factor for an effective immune response to RV vaccination. A recent study by Harris et al. demonstrated that antibiotics modestly affected the production of anti-RV IgA after oral RV vaccination, suggesting limited roles of microbiota in RV vaccine efficacy as changes in antibody levels were not substantial^28^. However, this study involved human adults from the Netherlands, a high-income region with typically high oral RV vaccine efficacy, which may explain the minimal impact of antibiotics treatment on RV-IgA levels due to less complex microbiota compositions^28–30^. Nonetheless, in low- and middle-income countries where microbiota environments are more diverse and complex, modulating host microbiota may significantly impact RV-specific antibody levels and the efficacy of oral RV vaccines, particularly in children.

This study has limitations. While Reg3β or Reg3γ appeared to mediate protection, we did not conduct dose-range study and thus could not rule out higher doses of one of them alone might also induce a similar protection. Similarly, the optimal doses of RANTES and Eotaxin need to be titrated to minimal effective doses in future studies to optimize protection while minimizing inflammation. This study employed a single IM RV inoculation rather than the multiple doses used in human vaccination. The EF and MPF mice models may not fully represent human gut microbiota complexity. Further, there is a lack of direct clinical validation, a short-term focus, and limited exploration of long-term effects. Proposed strategies, like combining oral and IM vaccinations, require rigorous clinical trials before implementation. More research is needed to understand the prevalence and composition of gut microbiota and their impact on rotavirus vaccine efficacy in children, especially in developing countries where rotavirus remains a major cause of severe diarrhea and a more effective vaccine is urgently needed.

## Experimental Model And Subject Details

### Mice

Female adult mice of C57BL/6 background aged 6–8 weeks were used in this study. EF and MPF C57BL/6 mice were obtained from Taconic Biosciences (Rensselaer, NY), and Rag1-KO mice were purchased from Jackson Laboratories (Bar Harbor, ME). The experiments the Reg3γ and Reg3β cytokine treatment on C57BL/6 mice (Jackson Laboratories) were approved by the Institutional Animal Care and Use Committee (IACUC), of the CDC and conducted in accordance with ethical guidelines for animal experiments and safety protocols. The other mice experiments conducted were approved by the IACUC of Georgia State University under animal protocol A20043. Upon arrival at the animal facilities, the mice were housed in autoclaved cages and provided with autoclaved chow (LabDiet 5010/5021) and water. All supplies were sterilized to ensure the mice remained in optimal health and consistent microbiota throughout the experiments.

### Euthanasia

Mice were euthanized via CO_2_ inhalation using the apparatus provided by the animal facility. During the CO_2_ inhalation euthanasia process, the animals were not anesthetized. The displacement rate was set at 20% of the chamber volume per minute and maintained for an additional 1–2 min following apparent clinical death. A secondary cervical dislocation was performed to assure the euthanasia. Cervical connection bones were physically checked to assure the cervical connection was disrupted.

### Viruses

A murine RV, EC strain, was used for all oral inoculation studies in mice. Cell-culture-adapted Wa (Human) and RRV (Simian) strains that were used for in vivo IM studies were propagated with MA-104 cells and titrated as previously described^31–33^. Wa and RRV, used in this study, are properties of the CDC. Dr. Harry Greenberg (Stanford, School of Medicine) kindly provided the EC strain of RV to CDC. Dr. Mary Estes (Baylor College of Medicine) kindly provided the EC strain of RV to GSU.

### Cell lines

The colorectal cancer cell lines HT-29 (HTB38™) and embryonic Rhesus monkey kidney tissue cell MA-104 (CRL2378.1™) were obtained from Biowhittaker. These cell lines were cultured using Dulbecco’s Modified Eagle Medium (DMEM) (Gibco, Carlsbad, CA) supplemented with 10% heat-inactivated fetal bovine serum (FBS) (Gibco) and 100 μg/mL neomycin (Sigma, St. Louis, MO). Proper cell culture techniques and protocols were followed during the study^31–33^.

## Method Details

### Virus infection

For acute in vivo RV infection, mice were gavaged with 100 μl 1.33% sodium bicarbonate (Sigma S5761), followed by oral inoculation of 10^4^–10^5^ SD_50_ or 10 SD_50_ (to mimic the scenario of oral RV vaccine non-responders) of RV, EC strain, in 100 μl phosphate-buffered saline (PBS)^12,33^.

### Fecal samples collections

Animals were individually placed in a clean cage without food and water for 1 h for fecal sample collection. After the collection, the animals were returned to their original cages^12^.

### Serum collections

For blood collection, the mice were anesthetized with isoflurane (3–5% induction/ 1–3% maintenance through a nose cone), and 200 μl of blood was collected from the submandibular site using a 6 mm animal lancet in serum separator tube (Greiner Bio-One 450472, Kremsmünster, Austria) in accordance with the animal protocol requirements. The collected blood samples were allowed to clot for 30 min at room temperature (RT) and then centrifuged at 2000 x g at 4 °C for 10 min to eliminate the clot. The remaining supernatant was then collected as serum^32^.

### Real-time PCR

Total mRNA was extracted from the mice ileum using TRIzol™ Reagent (Invitrogen™ Cat# 15596026, Waltham, MA) according to the manufacturer’s instructions. Gene expression levels were measured using a one-step qPCR kit (iTaq™ Universal SYBR® Green One-Step Kit, Bio-Rad, Hercules, CA) with the extracted total mRNA. Relative expression levels are calculated using the delta-delta Ct method. The primer sequences used are provided in Supplementary Materials Supplementary Fig. 7^34^.

### Measurement of RV antigen shedding

RV antigen shedding was measured using a sandwich ELISA as previously described in ref. ^32^. Fecal samples were obtained from mice and suspended in PBS at 100 mg/mL. The suspended samples were centrifuged at 10,000 g at 4 °C for 10 min, and the resulting supernatant was utilized for RV titration. A standard curve was created using serial dilutions of the EC strain of RV, which served as positive controls. A high-binding 96-well plate (Costar, 3590, Corning, NY) was coated with rabbit anti-RV antibody (Bio-rad, Hercules, CA, AHP1360, 1:1000 dilution in PBS) and stored overnight at RT. The coated plate was washed once with PBS-0.05% Tween20 (PBST) and blocked with 1% bovine serum albumin (BSA) in PBS for 1 h at RT. The plate was then washed once, and the prepared samples were loaded into the relative wells at a dilution of 1:10 in PBS and incubated for 1 h at RT. The plate was washed four times, and RV-specific antibody (PRF&L, Canadensis, PA, hyperimmune guinea pig anti-RRV serum, 1:1000 dilution in 1% BSA PBS) was added to each well. Following 1 h of incubation at RT, the plate was washed four times, and an anti-guinea pig-HRP-linked detection antibody (Jackson ImmunoResearch, 706-035-148, 1:10,000 dilution in 1% BSA PBS) was added to each well and incubated for 1 h at RT. The plate was washed four times, and 3,3’,5,5’-Tetramethylbenzidine (TMB) substrate solution (Invitrogen, 00420156) was added to each well. Signals were developed for 10 min and stopped using a stopping solution (0.16 M sulfuric acid), and absorbance was measured at 450 nm with a correction wavelength of 540 nm using a microplate reader (VersaMax, Molecular Devices, San Jose, CA).

### Quantification of mouse serum RV-specific IgG and fecal RV-specific IgA by ELISA

A high-binding 96-well plate was coated with 100 μl of rabbit hyperimmune sera against RRV at a dilution of 1:10,000 in carbonate/bicarbonate coating buffer (pH 9.6) and incubated overnight at 4 °C. The plate was washed three times with PBST and blocked with 200 μl of 5% skim milk (Difco™, Franklin Lakes, NJ) in PBS for 1 h at 37 °C. RRV (1 × 10^6^ focus-forming units per ml (ffu/ml) in blotto) was added to each well (100 μl/well) and incubated for 1 h at 37 °C. RV-immunized mouse serum and naïve mouse serum were used as positive and negative controls and were prepared in the same manner as the samples. The plate was then washed three times with PBST. The serially diluted mouse sera or mouse feces (100 μl) were added to the relative wells of the assay plate and incubated at 37 °C for 1 h. The plate was washed five times with PBST. The 100 μl of goat anti-mouse IgG-HRP (1:5000 dilution in 1% milk plus 0.5% normal rabbit serum, Sigma) or goat anti-mouse IgA-HRP (1:2000 dilution in PBS supplemented with 1% (wt/vol) skimmed milk, Difco™, Franklin Lakes, NJ) was added to each well of the plate. After 1 h of incubation, the plate was washed five times with PBST. TMB substrate was added to each well, and signals were allowed to develop for 10 min at RT. The reaction was stopped by adding 100 μl of 1 N HCl (Sigma). The absorbance was read at 450 nm (VersaMax microplate reader). The antibody titer in serum was defined as the reciprocal of the highest dilution that gave a mean OD greater than the cutoff value (0.1)^35^.

### Antibiotic treatment in drinking water

To prepare the antibiotic cocktail for consumption, ampicillin (1 g/L) (Sigma A6140), neomycin (1 g/L) (Sigma N6386), streptomycin (1 g/L) (Sigma S9137), and metronidazole (1 g/L) (Sigma M3761) were added to the drinking water. The experimental groups were provided with the antibiotic cocktail to consume one week before RV inoculations and were maintained on it throughout the whole experiment.

### Microneutralization assay for RV

RV micro-neutralization assay was performed as previously described in refs. ^36,37^. Briefly, the collected serum was heat-inactivated at 56 °C for 30 min. The RV (Wa) stock was activated with trypsin (15 µg/ml) at 37 °C for 1 h and a mock control was prepared. After inactivating and serially diluting the serum, it is combined with activated virus (1,200 FFU in 0.025 ml) at a 1:1 ratio. Following a one-hour incubation at 37 °C, MA104 cells are introduced to the serum: virus mixture and left to incubate for 3 days at 37 °C. Both the virus and mock control were added to wells with different serum dilutions. Then, the plate was fixed with 15 μl of 37% Formaldehyde at 4 °C for 30 min and blocked for each well. The antigen was identified using a polyclonal rabbit anti-WA serum and a colorimetric substrate. The neutralizing antibody titer was determined based on the highest serum dilution causing a 70% reduction in absorbance compared to the positive-control wells^37^.

### Influenza vaccination, challenge, and sera IgG ELISA

Influenza A/California/04/2009 (A/Cal) H1N1 virus was kindly provided by Dr. Richard Webby and amplified in 11-day old embryonated hen’s eggs. The sCal vaccine used in this study was prepared by inactivating A/Cal virus with formalin at a final concentration of 1:4000 (v/v) and then splitting with 1% triton x-100 as previously described in ref. ^17^. To evaluate the efficacy of the vaccines, EF WT mice were administered 10 µg of sCal vaccine by IM injection. Three weeks after inoculation, the mice were challenged with A/Cal virus at a dose of 3X LD_50_ (Lethal dose 50; 1X LD50 induces 50% lethal rate in EF WT mice) by intranasal administration. Mouse body weight and survival rates were monitored and recorded for a period of 3 weeks post-challenge. To determine the sCal-specific IgG in the serum samples, a 96-well plate was coated with 100 µL of inactivated A/Cal virus (20 µg/mL per well). Serially diluted serum samples (ranging from 1:100 to 1:12,500 in PBS) were added to the precoated wells. The relative levels of A/Cal-specific IgG were determined using anti-mouse IgG HRP (Southern Biotech, Birmingham, AL, USA), and signal development was achieved by adding TMB substrate (eBioscience, San Diego, CA, USA) for 10 min, followed by stopping the reaction using stopping solution (0.16 M sulfuric acid). Absorbance was measured at 450 nm, with a correction wavelength of 540 nm, using a microplate reader (VersaMax)^17^.

### 16S rRNA sequencing

Fecal DNA of xxx mice was extracted using DNeasy 96 PowerSoil Pro QIAcube HT Kit (Qiagen) and the region V3-V4 of 16S rRNA genes were amplified using the following primers: 341 F 5′TCGTCGGCAGCGTCAGATGTGTATAAGAGACAGCCTACGGGNGGCWGCAG-3′; 805 R 5′GTCTCGTGGGCTCGGAGATGTGTATAAGAGACAGGACTACHVGGGTATCTAATCC-3′. PCR products of each sample were purified using Ampure XP magnetic purification beads. An index PCR was performed to attach dual barcodes and Illumina sequencing adapters using Nextera XT Index kit (Illumina). Final PCR products was verified on 1.5% DNA agarose gel and quantified using Pico dsDNA assay (Invitrogen). An equal molar of each sample was combined and purified again using Ampure XP beads as the library. The library was diluted and spiked with 10% PhiX control (Illumina) and sequenced by Illumina iSeq™ 100 Sequencing System (2 x 150 bp). Demultiplexed fastq files were generated on the instrument. Sequencing data analysis was done in Qiime2 as previously described in ref. ^38^. Taxonomy was assigned based on Greengene 13.8 99% database. The raw 16S rRNA sequencing data generated in this study have been deposited in the NCBI Sequence Read Archive (SRA) under BioProject accession number PRJNA1247574.

### Serum cytokine assay

Serum cytokine profiles, including IL-1α, IL-1β, IL-2, IL-3, IL-4, IL-5, IL-6, IL-9, IL-10, IL-12 (p40), IL-12 (p70), IL-13, IL-17A, Eotaxin, G-CSF, GM-CSF, IFN-γ, KC, MCP-1 (MCAF), MIP-1α, MIP-1β, RANTES, and TNF-α were performed by using a Bio-Plex Pro Mouse Cytokine 23-plex Assay kit (#M60009RDPD, Bio-Rad) according to the manufacturer’s instructions.

### Virus preparation and recombinant proteins treatment experiment

Wa and RRV RV strains, were cultivated using MA-104 cells, and purified from cell culture supernatants by using CsCl gradient centrifugation. The virus was then inactivated in 62 °C water bath for 6 h and protein concentration was determined by protein assay kit (5000001, Bio-Rad, Hercules, CA)^22^. For the RV inoculation inhibition experiment, mice received IP injections of 200 μl of PBS with or without the recombinant Reg3β (5110-RG, bio-techne, Minneapolis, MN), Reg3γ (8189-RG, bio-techne), SAA1 (2948-SA, bio-techne, Minneapolis, MN), or Retnlb (NBP1-99306, bio-techne, Minneapolis, MN) at a dose of 10 μg each per 20 grams of body weight on 0- and 1-dpi. Following injections, the mice were orally challenged with the EC strain of RV. Fecal samples were collected daily until 10 dpi, and blood was collected at 21 dpi. For boosting IM RV inoculation efficacy experiment, mice were intramuscularly (IM) injected on the thigh muscle with 20 μl of PBS containing purified RV (Wa, 5 μg live or inactivated) mixed with or without RANTES (5 μg, 478-MR, bio-techne) or Eotaxin (2 μg 420-ME, bio-techne).

## Quantification And Statistical Analysis

Data were plotted using software GraphPad Prism (Version 8.0, Dotmatics, Boston, MA) and statistical significance was determined by 2-way ANOVA and unpaired Student’s t-test. Differences between experimental groups were considered significant at *p < 0.05, **p < 0.01, ***p < 0.001, and N.S., not significant.

## Supplementary information

Supplementary Material

## Data Availability

This paper does not report original code. Any additional information needed for the use of the data presented in this work paper is available from the lead contact upon request.

## Abbreviations

RV: Rotavirus
EF: excluded flora
MPF: murine pathogen free
SFB: Segmented filamentous bacteria
