# Supplementary material for: Gut microbiota modulate immune responses to orally and parenterally administered rotavirus in mice
Source: NPJ Vaccines. 2025 Apr 20;10:79. doi: 10.1038/s41541-025-01126-9 (PMC12009995; doi:10.1038/s41541-025-01126-9)
Supplement: Supplementary file 1 — Supplementary Material [file 41541_2025_1126_MOESM1_ESM.docx]

Supplementary Materials for

**Gut microbiota modulate immune responses to orally and parenterally administered rotavirus in mice**

Zhenda Shi^1,2,3^, Sung-Sil Moon^1^, Jun Zou^3^, Yanling Wang^3^, Noopur Bhatnagar^3^, Vu L. Ngo^3^, Xiaoqian Wang^1,2^, Houping Wang^1^, Theresa K. Bessey^1^, Jennifer J. Hull^1^, Yuhuan Wang^1^, Sang-Moo Kang^3^, Andrew T. Gewirtz^3^, and Baoming Jiang^1^

^1^ Division of Viral Diseases, National Center for Immunization and Respiratory Diseases, Centers for Disease Control and Prevention, Atlanta, GA, USA

^2^ Cherokee Nation Operational Solutions, Cherokee Federal, Atlanta, GA and Tulsa, OK, USA

^3^ Institute for Biomedical Sciences, Georgia State University, Atlanta, GA, USA

*Corresponding Authors: Dr. Zhenda Shi, E-mail: pst2@cdc.gov

Dr. Baoming Jiang, E-mail: bxj4@cdc.gov

**The PDF file includes:**

Supplementary Figure 1 to 7

Supplementary Figure 1. Related with Figure 2. Antibiotic treatment shifted the microbiota composition of MPF mice towards that of EF mice treated with and without antibiotics

Supplementary Figure 1. Related with Figure 1. Microbiota composition influences infection and antibody response to oral RV inoculation

EF and MPF mice were administered with or without antibiotics (Abx) cocktail in drinking water for one week. Fecal microbiota compositions were analyzed by using 16S-rRNA sequencing. Figure shows detailed 16s reads of different microbiota population.


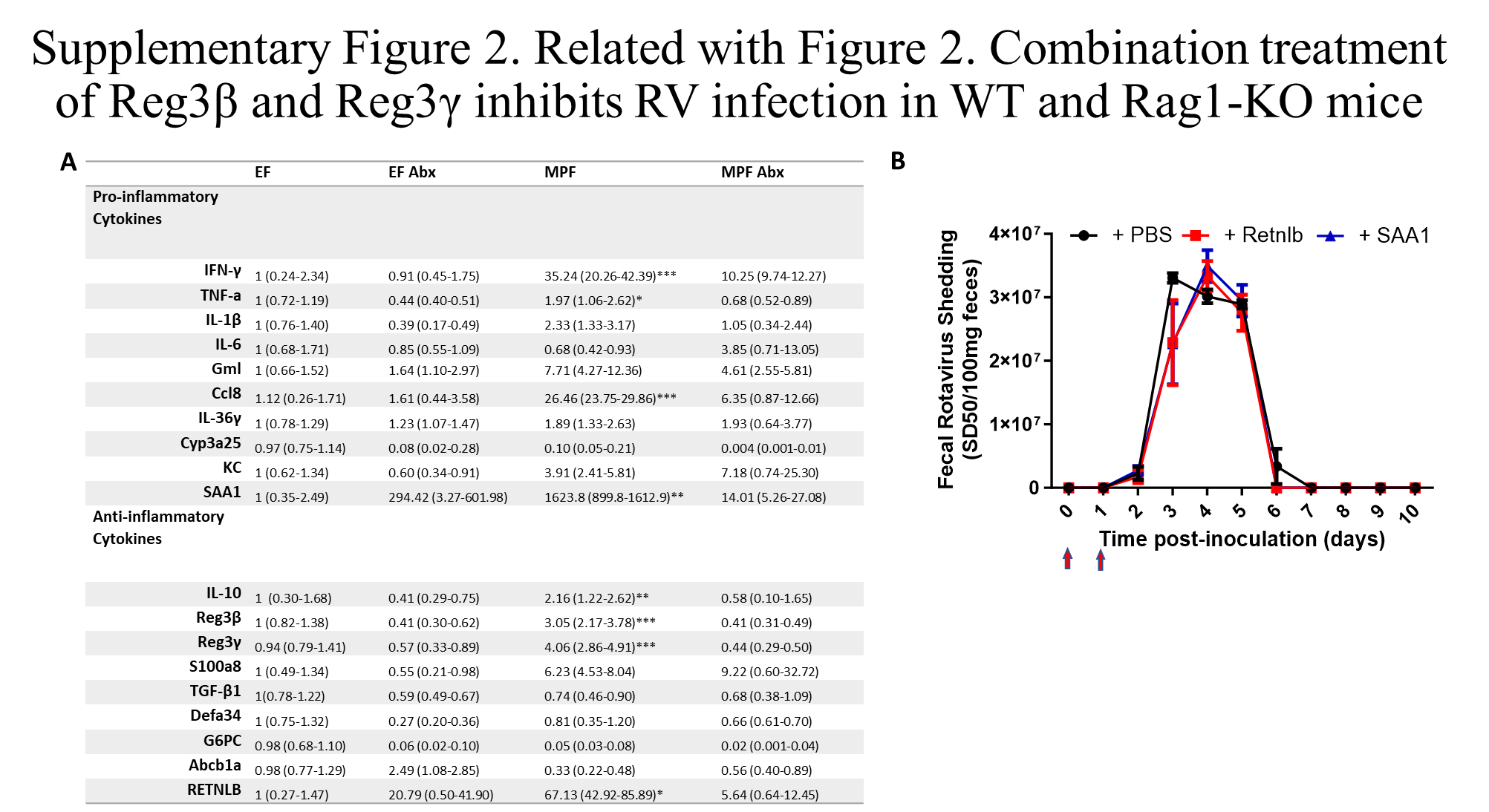


Supplementary Figure 2. Related with Figure 2. Combination treatment of Reg3β and Reg3γ inhibits RV infection in WT and Rag1-KO mice

**(A)** EF and MPF mice that were treated with or without antibiotics for 1-week, then ileum mRNA was extracted, and representative pro-inflammatory and anti-inflammatory cytokine expression levels were analyzed. Data are shown in relative mean and relative minimum to maximum expression levels. Data was analyzed by using T-TEST of MPF mice in comparison with the other 3 groups individually (n=4; *, p < 0.05, **, p < 0.01, ***, p < 0.001, determined by T-test).

**(B)** WT mice were intraperitoneally (IP) injected with PBS containing with or without Retnlb (10 µg) or SAA1 (10 µg) on the 0-dpi and 1-dpi (red arrow). The treated mice were challenged with RV orally on 0-dpi. Feces were collected daily for 10 days, and fecal RV antigen shedding was quantified by ELISA.


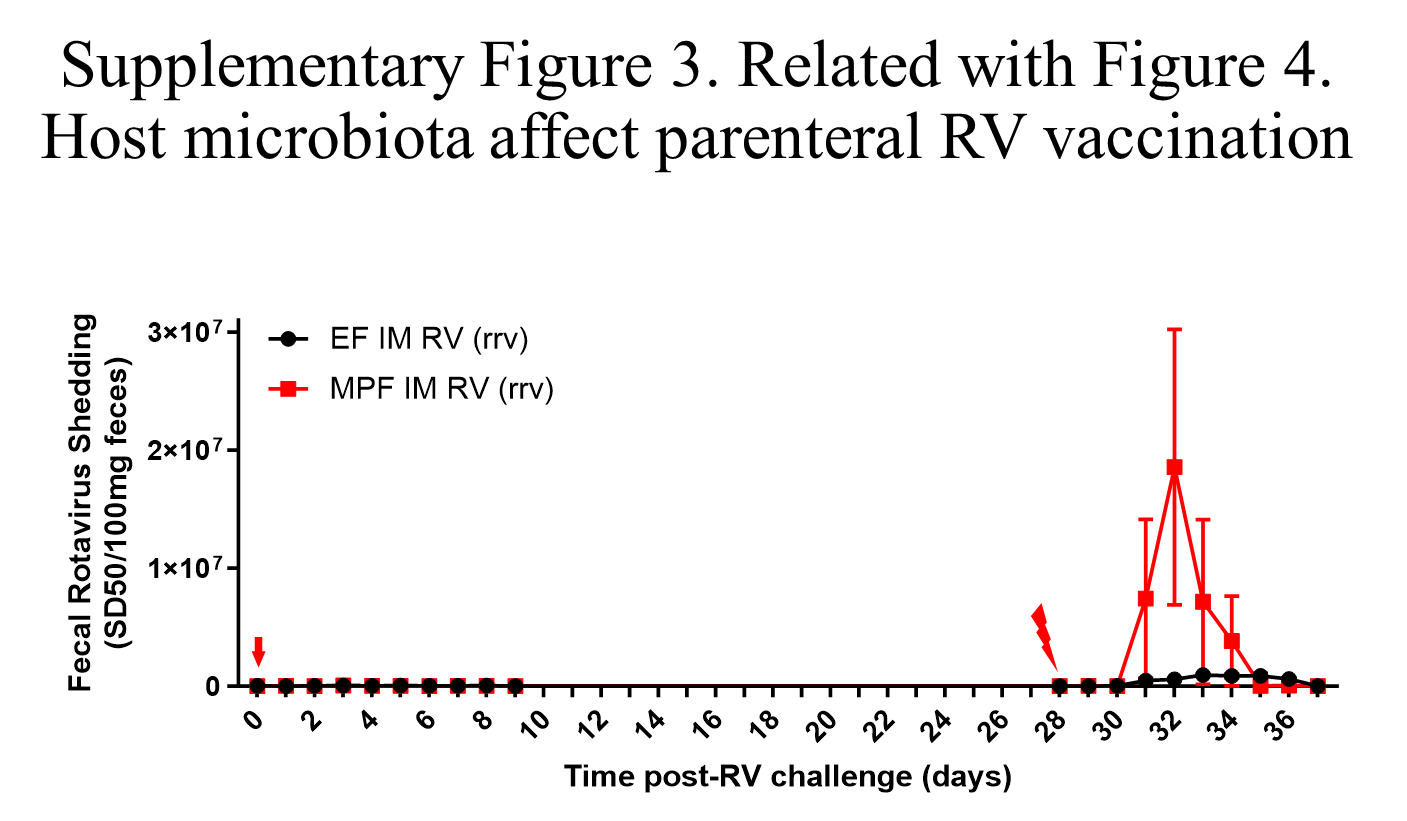


Supplementary Figure 3. Related to Figure 4. Host microbiota affect parenteral RV vaccination

EF or MPF mice received IM inoculation of RV (RRV strain 5 μg) on 0 dpi and challenged orally with RV (EC strain) on 30 dpi. Fecal samples were collected on indicated days, and fecal RV antigen shedding was quantified using ELISA. Data shown are means ± SEM, MPF IM RV differed EF IM RV group significantly (n= 4, p< 0.0001, two-way ANOVA)


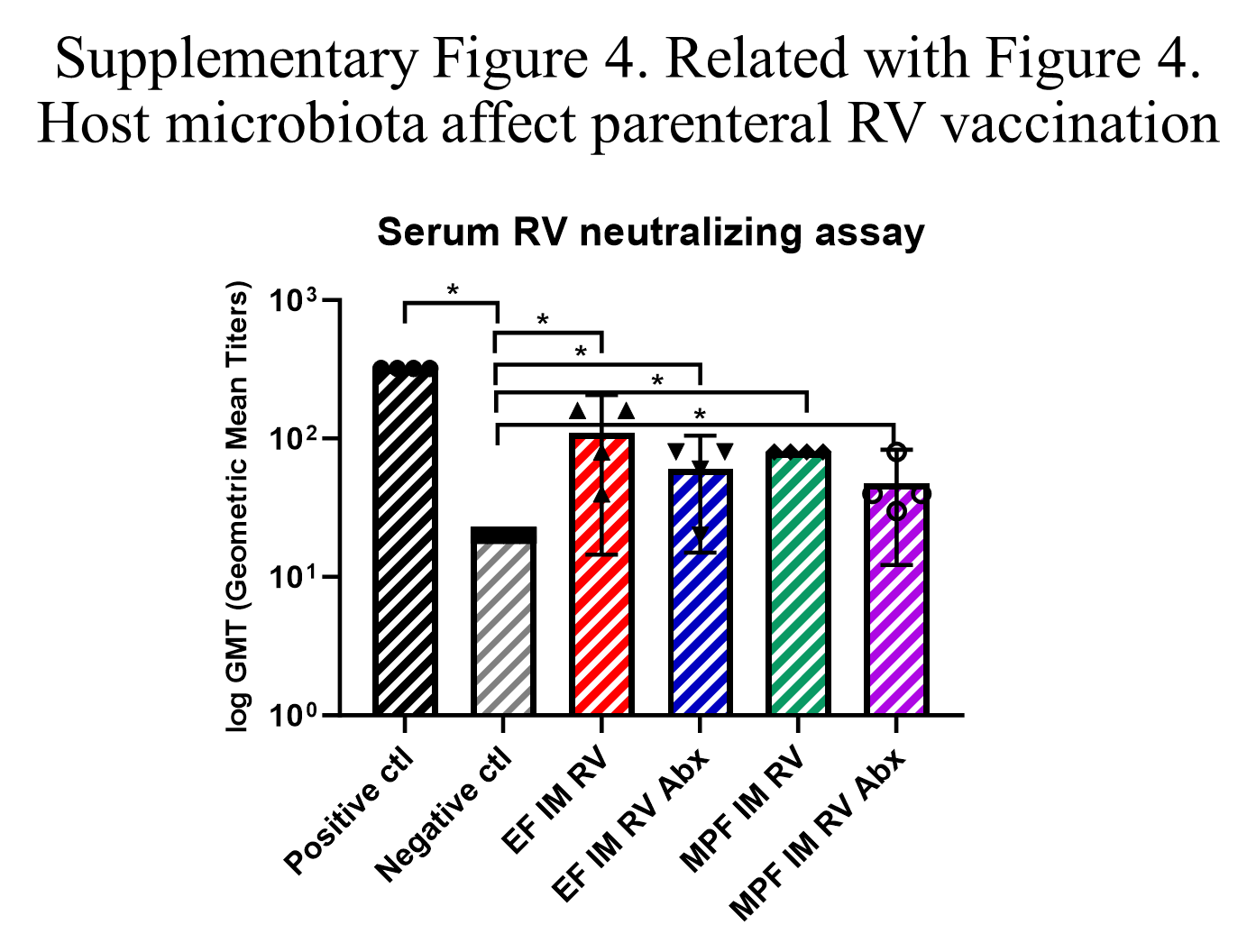
Supplementary Figure 4. Related to Figure 4. Host microbiota affect parenteral RV vaccination

EF and MPF mice were put on drinking water with or without antibiotics cocktail for 7 days before IM immunization of RV (Wa strain 5 μg) and maintained throughout the experiment. Serum was collected on 21-dpi, and RV neutralization activities were determined by microneutralization assay. The negative neutralization control differed from all the other groups significantly; no significance was found among the positive neutralization control, EF IM RV, EF IM RV Abx, MPF IM RV, and MPF IM RV Abx groups (data shown as GMT, ***, p < 0.001, n=4, determined by T-test).


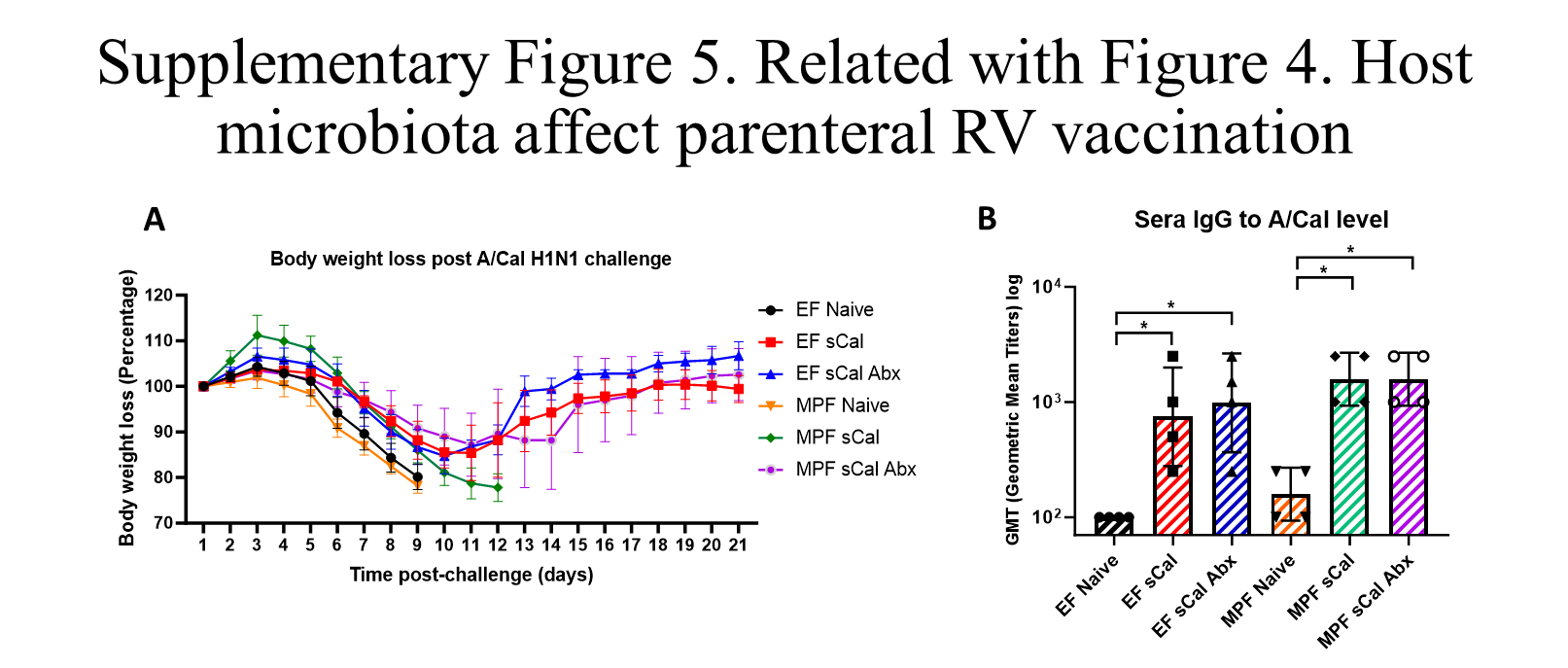
 Supplementary Figure 5. Related to Figure 4. Host microbiota affect parenteral RV vaccination

EF and MPF mice were put on drinking water with or without antibiotics cocktail for 7 days before IM immunization of sCal (split vaccine 10 μg) and maintained throughout the experiment. The mice were intranasally challenged with A/Cal (3X LD50) at 3-weeks post IM sCal immunization. Body weight changes were monitored for 21 days and serum sCal specific IgG were measured on day 21 dpi.

**(A)** Body weight changes post intranasal challenge of A/Cal. **(B)** Serum A/Cal H1N1-specific IgG levels.

Data shown in Fig. S5A are means ± SEM, EF sCal, EF sCal Abx, and MPF sCal Abx groups differed EF naïve, MPF naïve, and MPF sCal groups significantly, MPF sCal group differed all other groups significantly (n = 4, p < 0.0001, two-way ANOVA). Data shown in Fig. S5B are GMT, no significance was found among the four IM sCal immunized groups, but all differed the EF naïve and MPF naïve mice significantly (n = 4, * p<0.05, T-test). Each experiment was performed 2 times and yielded an identical pattern of results.


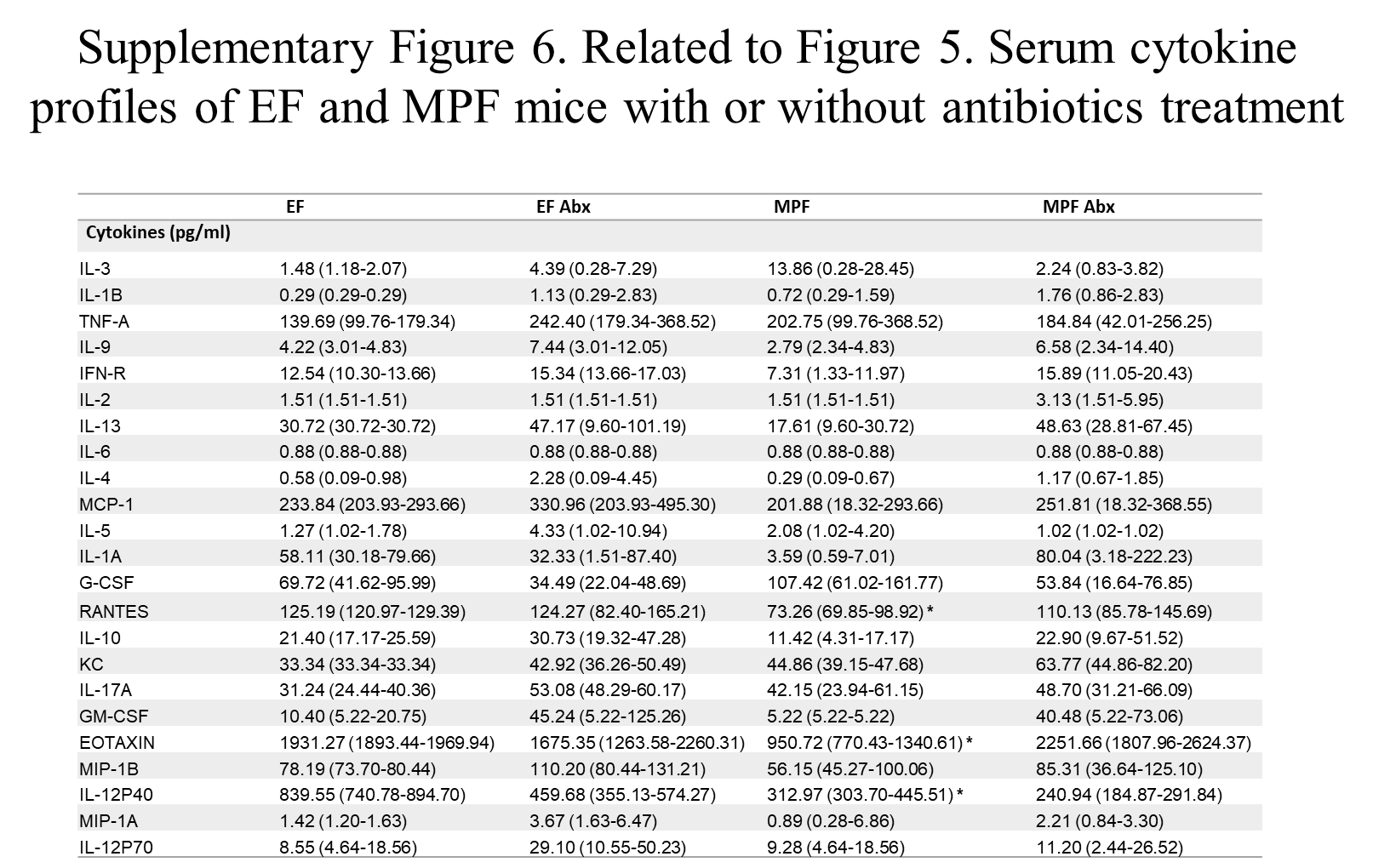
 Supplementary Figure 6. Related with Figure 5. Serum cytokine profiles of EF and MPF mice with or without antibiotics treatment

EF and MPF mice that were treated with or without antibiotics for 1-week, serum cytokine levels were analyzed using mice cytokine bio plex assay. Cytokine levels are shown in mean and minimum to maximum levels. Data are analyzed by comparing the cytokine levels of the MPF mice with the other 3 groups individually (n=4; *, p < 0.05, **, p < 0.01, ***, p < 0.001, determined by T-test).


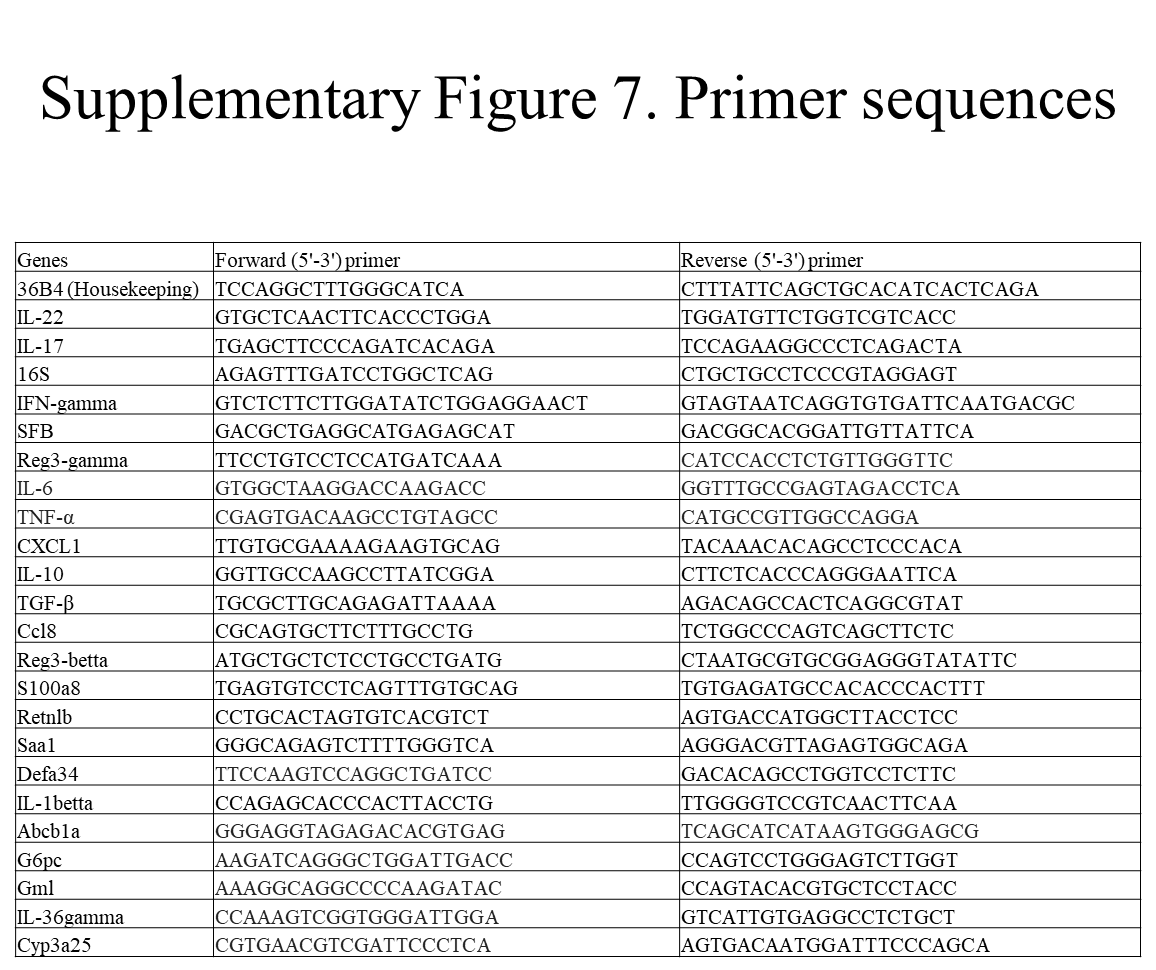
 Supplementary Figure 7. Primer sequences
